# A diagnostic signature derived from NK cell related genes in prostate cancer: insights from integrated scRNA-seq and bulk RNA-seq analyses with functional validation of KIT

**DOI:** 10.3389/fimmu.2026.1692792

**Published:** 2026-06-03

**Authors:** Zhengjun Chen, Fang Zhou, Jingzhi Tian, Qian Lv, Dong Wang, Shida Fan

**Affiliations:** Robotic Minimally Invasive Surgery Center, Sichuan Provincial People’s Hospital, School of Medicine, University of Electronic Science and Technology of China, Chengdu, China

**Keywords:** diagnostic model, immunotherapy, NK cell marker genes, prostate cancer, single-cell and bulk RNA-sequencing

## Abstract

**Background:**

Prostate cancer is one of the most common malignant tumors of the male genitourinary system. The impaired activity of natural killer (NK) cells observed in prostate cancer may contribute to immune evasion. This study aimed to develop robust NK cell-related diagnostic signatures.

**Methods:**

Based on NK related genes identified by scRNA-seq analysis, weighted gene co-expression network analysis, least absolute shrinkage and selection operator regression analysis, and machine learning algorithms were used to develop a novel diagnostic model. The expression of diagnostic genes was validated using tumor and adjacent normal tissues collected from prostate cancer patients. The biological functions of KIT as an NK cell related gene were further evaluated in prostate cancer cells.

**Results:**

A nine-gene NK cell-related diagnostic signature was developed, including *HSPD1*, *HSPE1*, *CLU*, *KIT*, *LAPTM4A*, *SLC18A2*, *TUBA4A*, *VWA5A*, and *ZFP36L1*. These genes were validated in independent datasets and showed strong predictive ability for prostate cancer diagnosis (AUC >0.8). Based on the expression profiles of these genes, nine compounds were identified that may influence drug sensitivity in prostate cancer. Furthermore, the expressions of *CLU*, *TUBA4A*, and *KIT* were successfully validated in collected prostate cancer tissue samples. Functional experiments demonstrated that KIT overexpression enhanced the cytotoxicity of NK-92 cells against prostate cancer PC-3 cells, inhibited cancer cell viability, cell migration and invasion, and increased the secretion of cytokines such as IFN-γ, Gzms-A, Gzms-B, and Perforin, as well as the degranulation marker CD107a.

**Conclusion:**

This study provides a new understanding of NK cell related gene signatures in prostate cancer diagnosis and highlights KIT as a promising candidate for future therapeutic investigation. Further research is needed to explore the mechanisms underlying the expression of these genes and their roles in the tumor microenvironment.

## 1 Introduction

Prostate cancer is a common malignancy of the male urinary system globally (1). It is estimated that from 2020 to 2040, the global incidence of prostate cancer will continue to rise, with an increase of approximately 85% (2). Although prostate-specific antigen (PSA) testing and multiparametric magnetic resonance imaging (mpMRI) are widely used clinical tools for prostate cancer monitoring, their specificity remains limited (3). The five-year survival rate for patients with early-stage prostate cancer is around 99%, whereas it drops drastically to only 31% for those with metastatic disease (4). Androgen deprivation therapy (ADT) is important for prostate cancer treatment; however, most patients eventually progress to castration-resistant prostate cancer (CRPC), which significantly limits further therapeutic options and efficacy (5, 6). Therefore, elucidating the molecular mechanisms underlying prostate cancer and identifying effective and reliable genetic features are critical for improving diagnostic accuracy and advancing therapeutic strategies.

The tumor immune microenvironment (TIME) is composed of immune cells, tumor cells, stromal cells, and other components. The complex interactions among these elements play a crucial role in regulating tumor growth, metastasis, and response to immunotherapy (7, 8). Natural killer (NK) cells, a key component of the innate immune system, are indispensable in anti-tumor immunity, capable of directly lysing tumor cells and enhancing T cell-mediated responses (8, 9). However, NK cell plasticity may also lead to pro-tumor phenotypes under certain conditions (10). Preclinical mouse models of prostate cancer have demonstrated that boosting NK cell-mediated tumor clearance represents a promising alternative therapeutic strategy (11). Furthermore, men with low NK cell activity have a fivefold higher likelihood of being diagnosed with prostate cancer upon biopsy, suggesting that NK cell-related features may serve as valuable biomarkers for early detection (12).

Single-cell RNA sequencing (scRNA-seq) facilitates the characterization of the molecular features of immune cell populations within the TIME, providing a powerful approach for identifying functional biomarkers (13, 14). Integrating scRNA-seq with bulk RNA-seq data enables a more comprehensive understanding of gene expression profiles (15–17). Previous studies have highlighted the molecular complexity of prostate cancer and demonstrated the feasibility of applying multi-omics approaches to identify potential biomarkers (18, 19). In this study, we employed multiple bioinformatics analyses to identify NK cell related genes and construct a diagnostic model for prostate cancer. We subsequently validated the biological function of KIT as an NK cell related gene in prostate cancer cells. Overall, this study provides valuable insights into the development of potential diagnostic biomarkers and therapeutic targets for prostate cancer.

## 2 Methods and materials

### 2.1 Data collection

scRNA-sequencing data from three treatment-naïve patients (Gleason score 9) with prostate cancer in GSE153892 were acquired from the Gene Expression Omnibus (GEO, https://www.ncbi.nlm.nih.gov/) database to screen for NK cell related genes. These data were specifically selected after sorting CD45^+^ cells (immune cells), because the high cell purity obtained through this process makes them highly suitable for TIME studies. The Cancer Genome Atlas (TCGA) bulk tumor transcriptomic data in count or fragments per kilobase million (FPKM) form and clinical information of 497 prostate cancer and 52 adjacent tissues were downloaded from UCSC Xena (https://xenabrowser.net/) as a training cohort. GSE21034, including 29 normal adjacent samples and 131 prostate cancer samples, and GSE134051, including 39 benign prostate hyperplasia and 216 prostate cancer samples, were selected for validation.

### 2.2 scRNA-seq data analysis

Seurat and SingleR packages were used to analyze the scRNA-seq data. To acquire high-quality scRNA-seq data, the filtration criteria were processed as follows: only genes that were expressed in at least three single cells were retained, cells that expressed fewer than 200 genes were discarded, and cells with more than 5% mitochondrial genes were also discarded. After standard screening, 10, 444 cells were obtained. Next, the “NormalizeData” package was used for normalization. The top 20 principal components (PCs) were obtained using principal component analysis (PCA) based on the top 1, 500 highly variable genes. The T-distributed random neighborhood embedding (tSNE) algorithm was employed to dimensionally reduce the top 20 PCs, followed by cell clustering analysis. Different cell clusters, including those related to NK cells, were identified and annotated using the singleR package, focusing on the composition patterns of the marker genes. Subsequently, the limma package in R was used to annotate differentially expressed genes (DEGs) across the NK cell clusters and other cell clusters. NK cell cluster related genes were selected from NK-related clusters based on |log_2_FC| > 1 and p-value < 0.05.

### 2.3 Estimation of cell infiltration in the tumor microenvironment

A single-sample gene set enrichment analysis (ssGSEA) algorithm was used to quantify the relative abundance of TIME cell infiltration in prostate cancer. Each TIME-infiltrating immune cell marker gene set was acquired from Charoentong’s study (20). The Wilcoxon rank-sum test was used to compare the infiltration level of immune cells between adjacent and prostate cancer tissues. This nonparametric test is suitable for comparing two independent samples when the data distribution is not normally assumed (21).

### 2.4 Identification of DEGs in TCGA prostate cancer

DEGs between prostate cancer and adjacent control samples were identified using the limma R package, with a threshold of |log_2_FC| > 0.5 and FDR < 0.05. The heatmap and volcano map were drawn using the R packages pheatmap and ggplot, respectively.

### 2.5 Weighted gene co-expression network analysis

WGCNA is a typical systematic biological algorithm used to describe the correlation patterns among gene expression profiles and to construct gene co-expression networks. The R package ‘WGCNA’ was used to analyze the co-expression network of mRNAs with the top 25% coefficient of variation and to construct a scale-free gene co-expression network. The “hclust” function was used to remove outliers, and “pickSoftThreshold” in WGCNA was used to determine the optimal soft threshold (β). Subsequently, the co-expression similarity was calculated based on the soft threshold, and adjacency was computed. Modules were determined based on hierarchical clustering and dynamic tree cutting. Finally, genes with similar expression patterns were clustered together, and modules were divided using default parameters according to the “cutreeDynamic” function. Because the modules identified by the dynamic tree-cutting algorithm may be similar, we merged similar modules according to a soft threshold of 0.25.

### 2.6 Identification of core genes

Based on the WGCNA, modules related to NK cells and prostate cancer were regarded as key modules. The intersection of genes from this WGCNA-derived key module, NK cell cluster related genes, and DEGs yielded key genes associated with NK cell alterations during prostate cancer development and progression. Subsequently, Spearman correlation analysis was used to evaluate the correlation between key genes and NK cells, and the genes with p-value < 0.05 and |Correlation| > 0.3 were defined as the core genes. Functional enrichment analysis of core genes was performed using the DAVID tool with a p-value < 0.05, including Kyoto Encyclopedia of Genes and Genomes (KEGG) and Gene Ontology (GO) analyses.

### 2.7 Feature selection and model construction

Machine learning algorithm modeling was used to identify diagnostic genes for prostate cancer. First, least absolute shrinkage and selection operator (LASSO) regression analysis was performed using the R software “glmnet” package to screen out the diagnostic genes from the core genes. Second, the “randomforest” R package was used to rank the importance of each gene selected by LASSO according to the value of the average decreasing accuracy. Subsequently, the random forest algorithm and 10-fold cross-validation method were used to obtain the optimal number of diagnostic genes. Three classification models were established: support vector machine (SVM) model, random forest (RF) model, and decision tree (DT) model. The diagnostic capabilities of the three models were evaluated using receiver operating characteristic (ROC) curves.

### 2.8 Competitive endogenous RNA regulatory network of diagnostic genes

Using the ENCORI (http://starbase.sysu.edu.cn/index.php), TargetScanHuman 7.2 (https://www.targetscan.org/vert_72/), and miRDB (https://mirdb.org/) databases, miRNAs potentially targeting the diagnostic genes were predicted. Differentially expressed miRNAs (DEmiRNAs) between prostate cancer and control samples were identified from the GSE21036 dataset using the criteria of padj < 0.05 and |log_2_FC| > 0.1. DEmiRNAs that were negatively correlated with the diagnostic genes were selected. Subsequently, lncRNAs targeting these DEmiRNAs were predicted using the ENCORI database, with a screening threshold of padj < 0.05. Differentially expressed lncRNAs (DElncRNAs) were then identified from the TCGA prostate cancer dataset, and those negatively correlated with the selected DEmiRNAs were further screened. Finally, the ceRNA network was constructed based on DElncRNAs-DEmiRNAs-mRNAs regulatory relationships. Because ceRNA regulation is influenced by multiple factors (22), relatively inclusive thresholds were applied to avoid missing potential regulatory non-coding RNAs and to enable the preliminary construction of the ceRNA network.

### 2.9 Drug sensitivity analysis

The CADSP platform (https://smuonco.shinyapps.io/CADSP/) was used to assess drug sensitivity based on gene expression. It integrates 177 GEO datasets, 33 TCGA datasets, drug sensitivity information from the GDSC database, and gene perturbation data from GPSAdb (23). It also provides tools for data analysis and visualization using R (v.4.1.3). For each of the diagnostic genes, samples in the TCGA prostate cancer cohort were divided into high and low expression groups. We then compared the IC50 values of compounds between groups to identify drugs with significantly different sensitivities across both expression groups. The three-dimensional structures of the diagnostic gene–encoded proteins and small-molecule compounds were obtained from the RCSB Protein Data Bank and PubChem databases, respectively. Molecular docking was subsequently performed using CB-Dock2 (https://cadd.labshare.cn/cb-dock2/) to predict the lowest binding energies between candidate drugs and the diagnostic genes. CB-Dock2 integrates cavity detection, molecular docking, and template-based fitting to accurately predict potential binding sites. A blind docking strategy was applied, which is widely used in computer-aided drug discovery. Compared with other blind docking approaches, CB-Dock2 has been reported to improve docking success rates by approximately 16%-30% (24).

### 2.10 Real-time qPCR analysis

Six prostate cancer tissue samples and seven prostate cancer adjacent tissue samples were subjected to Real time qPCR validation. Written informed consent was obtained from all patients. Total RNA was extracted from the samples using the TRIzol reagent. FastQuant cDNA (TIANGEN, KR106) was used to synthesize cDNA. SuperReal PreMix Plus (TIANGEN, FP205) was used to amplify target RNA using a real-time PCR system (Applied Biosystems, ABI7300). The change in target gene expression was calculated using the 2^-ΔΔCt^ method. Primer sequences are shown in **Supplementary Table 1**.

### 2.11 Immunohistochemical and immunofluorescence analysis

Twenty human paraffin wax-embedded tissue samples were collected, including ten prostate cancer-adjacent tissue samples and ten prostate cancer tumor tissue samples. Embedded samples were sliced using a microtome to obtain 4 μm serial sections. Xylene was used for deparaffinization, and an ethanol/water gradient series was used to rehydrate the sections. The sections were infiltrated with a closed permeable solution (BOSTER, AR0150) at room temperature for 30 min. Tissue slides were immersed in 0.01 M sodium citrate buffer solution pH 6 (ZSGB-BIO, ZLI-9064) and then boiled in a microwave for antigen retrieval. Serial tissue sections from the same cases were incubated for 60 min at room temperature and then overnight at 4°C with the following primary antibodies: Pan-CK (ZSGB-BIO, ZM-0069, ready-to-use), CD3 epsilon Rabbit mAb (ZEN-BIO, R23808, 1:100), NCR1/NKp46 Rabbit mAb (ZEN-BIO, R51204, 1:50), c-Kit Mouse Monoclonal Antibody (Affinity, BF8286, 1:100) and Anti-CD56 (bioss, bs-0805R, 1:200). After several washes with phosphate-buffered saline (PBS), the tissue sections were incubated for 30 min at 37°C with the following secondary antibodies: goat anti-rabbit IgG H&L (MDL, MD912533, 1:200) and goat anti-mouse IgG H&L (MDL, 1:200). A DAB kit (ZSGB-BIO, ZLI-9017) was used for color development, and a hematoxylin staining solution (ZSGB-BIO, ZLI-9609) was used for dyeing. Images were captured using an optical microscope (Phenix, PH-XDS5). IHC scores were calculated using the product of the percentage of positive cells and staining intensity. The percentage of positive cells was scored as follows: 0, none; 1, 10-50%; 3, 50-80%; and 4, >80%. Staining intensity was graded as: 0, negative; 1, light brown; 2, brown; and 3, dark brown.

IF labeling was performed on the same samples used for IHC evaluation. Antigens were extracted using a heat-induction method. After several washes with PBS and serum blocking, the tissue sections were incubated for 60 min overnight at 4°C with the following primary antibodies: c-Kit Mouse Monoclonal Antibody (Affinity, BF8286, 1:200) and Anti-CD56 (bioss, bs-0805R, 1:200). Tissue sections were incubated for 60 min at 37°C with the following secondary antibodies: goat anti-rabbit IgG H&L (1:200) and goat anti-mouse IgG H&L (1:200). DAPI (Beyotime, C1005) was added, and the cells were incubated for 10 min in the dark. Tablets were sealed with an anti-fluorescence quencher and immediately observed under a fluorescence microscope (Nikon, ELCIPSE-CI). Statistical analyses were performed using GraphPad Prism software (v10.1.2, USA), and comparisons between cancer and adjacent tissue groups were conducted using the Wilcoxon paired test.

### 2.12 Cell culture and transfection

The human prostate cancer cell line (PC-3, Procell, CL-0185) and NK-92 cell line (Procell, CL-0530) were purchased from Wuhan Pricella Biotechnology Co., Ltd. PC-3 was cultured in complete medium (Procell, CM-0185) containing 10% fetal bovine serum (FBS) at 37°C in a 5% CO_2_ environment. NK-92 was cultured in complete medium supplemented with IL-2 (Procell, CM0530). The same IL-2 concentration was used across all experimental groups. To construct KIT-overexpressing NK-92 cells, NK-92 cells were transfected with the KIT overexpression plasmid (synthesized by BioMed, China) using Lipo8000™ (Beyotime, C0533) in 6-well plates and incubated for 48 hours before subsequent analyses. In subsequent experiments, NK-92 cells (1×10^5^) were co-cultured with PC-3 cells (2×10^4^) at an effector-to-target ratio of 5:1 to evaluate the effects of KIT overexpression on NK cell degranulation and tumor cell cytotoxicity.

### 2.13 Enzyme linked immunosorbent assay

The concentrations of the cytokines and chemokines IFN-γ, Gzms-A, Gzms-B, and Perforin were evaluated to assess the functional outputs of degranulation using a commercial ELISA-based kit (SINOVAC, F0033-B; F0431-B; F0430-B; F1149-B) in the NK-92+PC-3, NK-92-OE-KIT+PC-3, and NK-92-OE-NC+PC-3 (vector control) groups. Following the manufacturer’s instructions, the standard product was diluted; the sample was added to the enzyme plate hole, incubated at 37°C for 30 min, and washed with washing solution several times; and the color developer was added. Absorbance was measured using a spectrophotometer at 450 nm as the primary wavelength.

### 2.14 Surface marker analysis by flow cytometry

To evaluate the killing ability of NK cells, CD107a expression was detected by flow cytometry in the NK-92+PC-3, NK-92-OE-KIT+PC-3, and NK-92-OE-NC+PC-3 (vector control) groups. Cells were centrifuged at 500 × *g* for 5 min, and the supernatant was discarded and added to the medium for resuspension and preparation of a single-cell suspension. The cell concentration was adjusted to 1 × 10^6^ cells per 100 μL, and mouse anti-human CD107a/PE antibody (Bioss, bsm-30204M-PE, 10 µL/Test) was added and incubated for 30 min. The cell suspension was then centrifuged at 500 rpm for 5 min, the supernatant was discarded, and the cells were washed with PBS. Finally, 200 μL of PBS re-suspension cells was added and detected by flow cytometry (Agilent, NovoCyte).

### 2.15 Cell migration and invasion

To investigate the effects of KIT-overexpressing NK-92 cells on the migration and invasion of PC-3 cells, a Transwell chamber assay (Corning, 3422) was performed. The experiment included four groups: PC-3 (negative control), NC-92+PC-3, NK-92-OE-KIT+PC-3, and NK-92-OE-NC+PC-3. Briefly, complete medium (500 µL) was added to the lower chamber, and serum-free medium containing 2 × 10^5^ mixed cells (NK-92 and PC-3) was added to the upper chamber. After 48 h, the cells that remained in the upper chamber were discarded, and those that migrated to the lower chamber were stained with crystal violet staining solution (Solarbio, G1063).

Diluted Matrigel (100 µL) was added to the upper chamber and incubated at 37°C until the Matrigel solidified. Complete medium (500 µL) was added to the lower chamber, and serum-free medium containing 2 × 10^5^ mixed cells was added to the upper chamber. After 48 h, the cells remaining in the upper chamber were discarded. The stained cells were counted under a light microscope (Phenix, PH-XDS5).

### 2.16 Flow cytometry-based apoptosis detection

A Cell Cycle and Apoptosis Analysis Kit (4A Biotech, FXP021; Solarbio, CA1020) was used to detect apoptosis in four groups. The cells were digested with ethylenediaminetetraacetic acid-free pancreatic enzymes and centrifuged at 1000 rpm for 5 min for precipitation. The cells were resuspended in precooled PBS at 4°C, and the supernatant was removed after centrifugation. The cell concentration was adjusted to 1-5 × 10^6^/mL using a buffer solution. The cell suspension was mixed with 5 µL of Annexin V/FITC and incubated at room temperature in the dark for 5 min. Finally, propidium iodide (PI) was added for staining and analyzed using flow cytometry.

### 2.17 CCK-8 assay for cell viability detection

A cell Counting Kit-8 (CCK-8; APEXBIO, K1018) assay was used to assess cell viability in four groups. PC-3 cells cultured alone were used as the negative control, while cell-free culture medium served as the blank control. CCK-8 reagent was added according to the manufacturer’s instructions, and absorbance was measured at 450 nm. Cell proliferation was calculated using the following formula: cell proliferation (%) = (experimental group − blank control)/(negative control − blank control) × 100%.

## 3 Results

### 3.1 Identification of NK cell cluster related gene expression profiles

**Figure 1:**
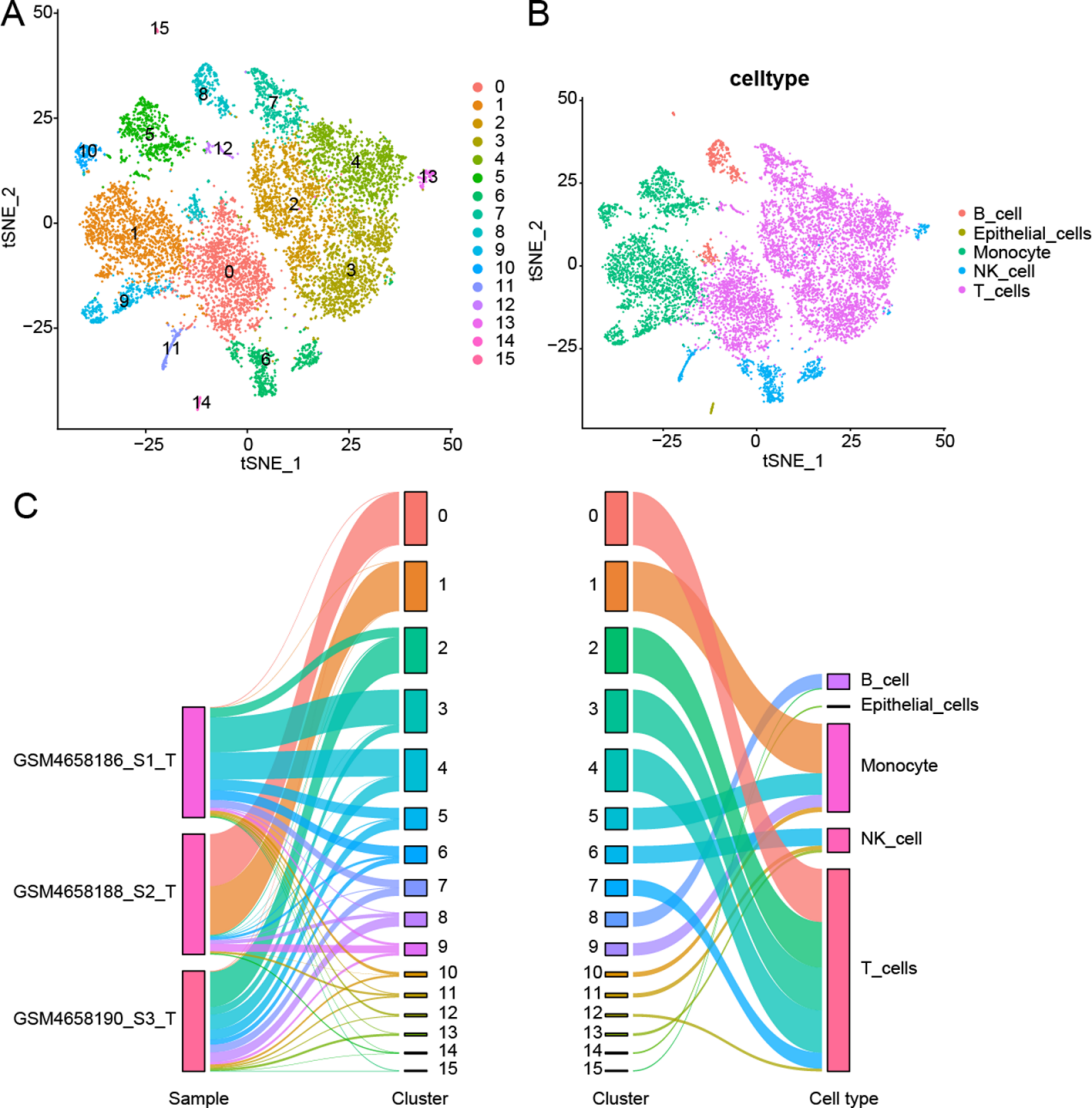
Identification of NK cell related genes in prostate cancer. **(A)** t-SNE plot categorized by clusters. **(B)** t-SNE plot categorized by cell types. **(C)** Sankey diagram illustrating the distribution of cell clusters across samples and their corresponding cell types.

ssGSEA showed that the infiltration level of NK cells in the prostate cancer group was markedly lower than that in the adjacent control group (**Supplementary Figure 1A**). This finding indicates a diminished presence of NK cells within the tumor microenvironment, potentially indicating reduced functionality. Additionally, the correlation analysis showed a strong positive correlation between NK cells and various immune cells (**Supplementary Figure 1B**), suggesting that NK cells may contribute to the antitumor immune response by interacting with other immune cells. From the scRNA-seq dataset GSE153892, we obtained 10, 444 qualified cells from three primary prostate cancer samples, along with expression data for 17, 555 genes for subsequent analysis. Based on the dimensionality reduction and clustering analysis, 10, 444 cells were grouped into 16 distinct clusters and classified into five major cell types using the t-SNE algorithm (**Figures 1A, B**). Then, clusters 6, 11, and 13 were annotated as NK-cells subpopulations (**Figure 1C**). Finally, we identified 106 NK cell cluster related genes with |log_2_FC| > 1 and p-value < 0.05.

### 3.2 Identification of core genes

**Figure 2:**
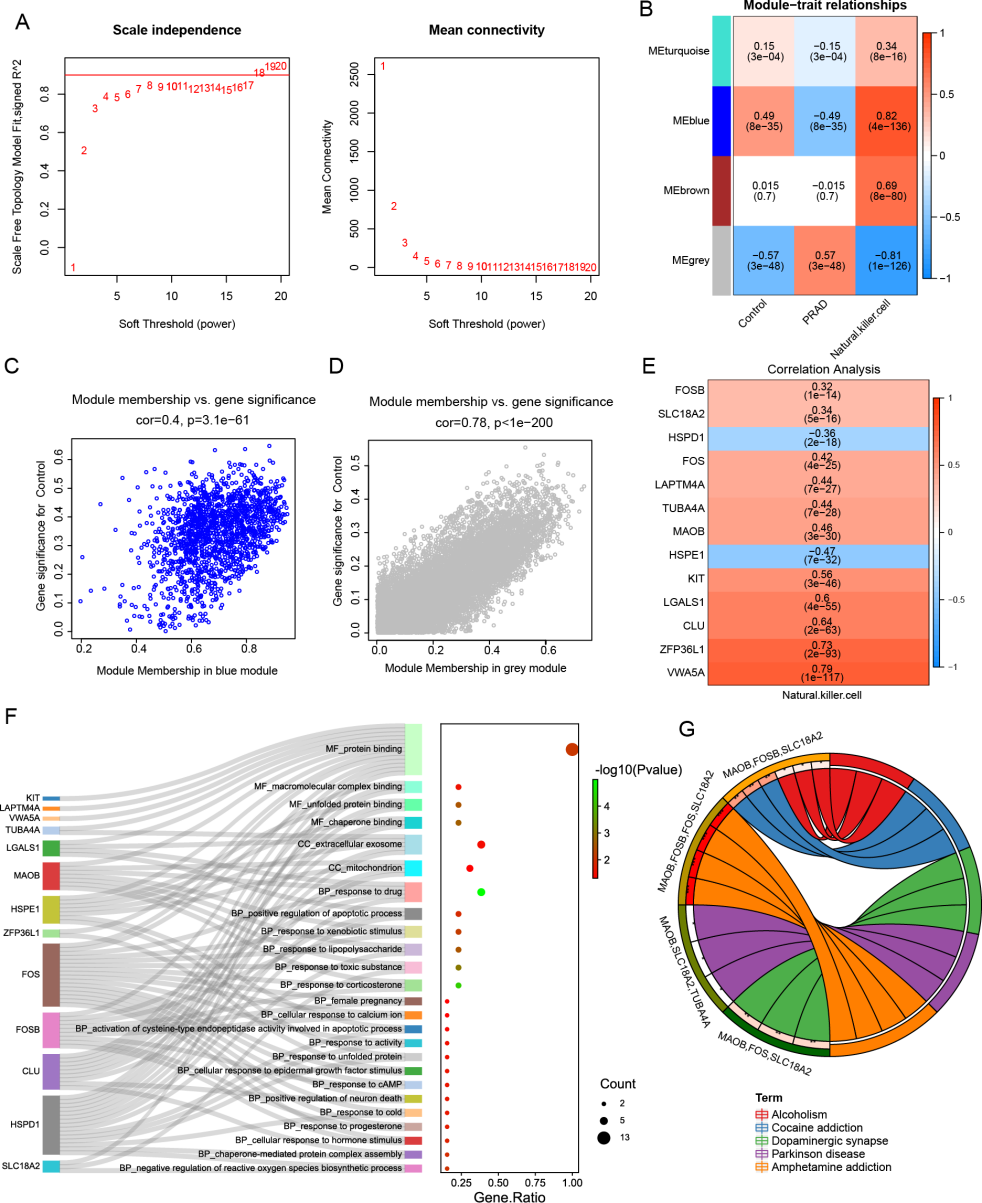
Identification of core genes associated with NK cells. **(A)** Scale-free fitting index of different soft threshold power (β) and mean connectivity of various soft threshold power. **(B)** Heatmap showing the correlation between prostate cancer (PRAD), NK cells and different gene modules. **(C)** Scatter plot representing the module membership and gene significance for the blue module. **(D)** Scatter plot representing the module membership and gene significance for the gray module. **(E)** Heatmap showing the correlation analysis between 13 core genes and NK cells. **(F)** Sankey diagram representing the GO enrichment results of the core genes. **(G)** Circular diagram displaying the KEGG enrichment results of the core genes.

Under the standard of |log_2_FC| > 0.5 and FDR < 0.05, there were 2, 159 DEGs between prostate cancer and adjacent control samples, including 993 upregulated DEGs and 1, 166 downregulated DEGs (**Supplementary Figure 2**). A scale-free network was constructed using WGCNA (β = 18, **Figure 2A**). Phenotypic data from prostate cancer and NK cell score (as determined by ssGSEA) were used to evaluate their correlations with the gene co-expression modules (**Figure 2B**). The blue module was positively correlated with NK cells (r = 0.82, p = 4e-136), and the grey module was negatively correlated with NK cells (r = 0.81, p = 1e-126). A total of 1, 566 genes were found in the blue module (**Figure 2C**) and 8, 932 genes were found in the grey module (**Figure 2D**).

Subsequently, 21 overlapping genes were identified by intersecting NK cell cluster related genes, DEGs between prostate cancer and adjacent tissues, and genes from two WGCNA modules (blue and grey). These genes include *CLU*, *LAPTM4A*, *SLC18A2*, *VWA5A*, *KIT*, *SPINK2*, *TUBA4A*, *SOX4*, *HSPD1*, *ZFP36L1*, *HSPE1*, *SPON2*, *MAOB*, *CA2*, *LGALS1*, *HSPB1*, *SAT1*, *FOS*, *MT1X*, *NR4A1*, and *FOSB*. Furthermore, Spearman correlation analysis revealed that 13 of the 21 genes (*FOSB*, *SLC18A2*, *HSPD1*, *FOS*, *LAPTM4A*, *TUBA4A*, *MAOB*, *HSPE1*, *KIT*, *LGALS1*, *CLU*, *ZFP36L1*, and *VWA5A*) were significantly correlated with NK cells with p <0.05 and |r| >0.3 (**Figure 2E**). GO analysis showed that the 13 core genes were mainly enriched in a series of key biological events, such as mitochondria, extracellular exosomes, protein binding, response to xenobiotic stimulus, positive regulation of the apoptotic process, and response to drugs (**Figure 2F**). KEGG pathway enrichment results showed that the 13 core genes were mainly involved in dopaminergic synapse pathways (**Figure 2G**).

### 3.3 Identification of the optimal diagnostic NK cell related genes for prostate cancer

**Figure 3:**
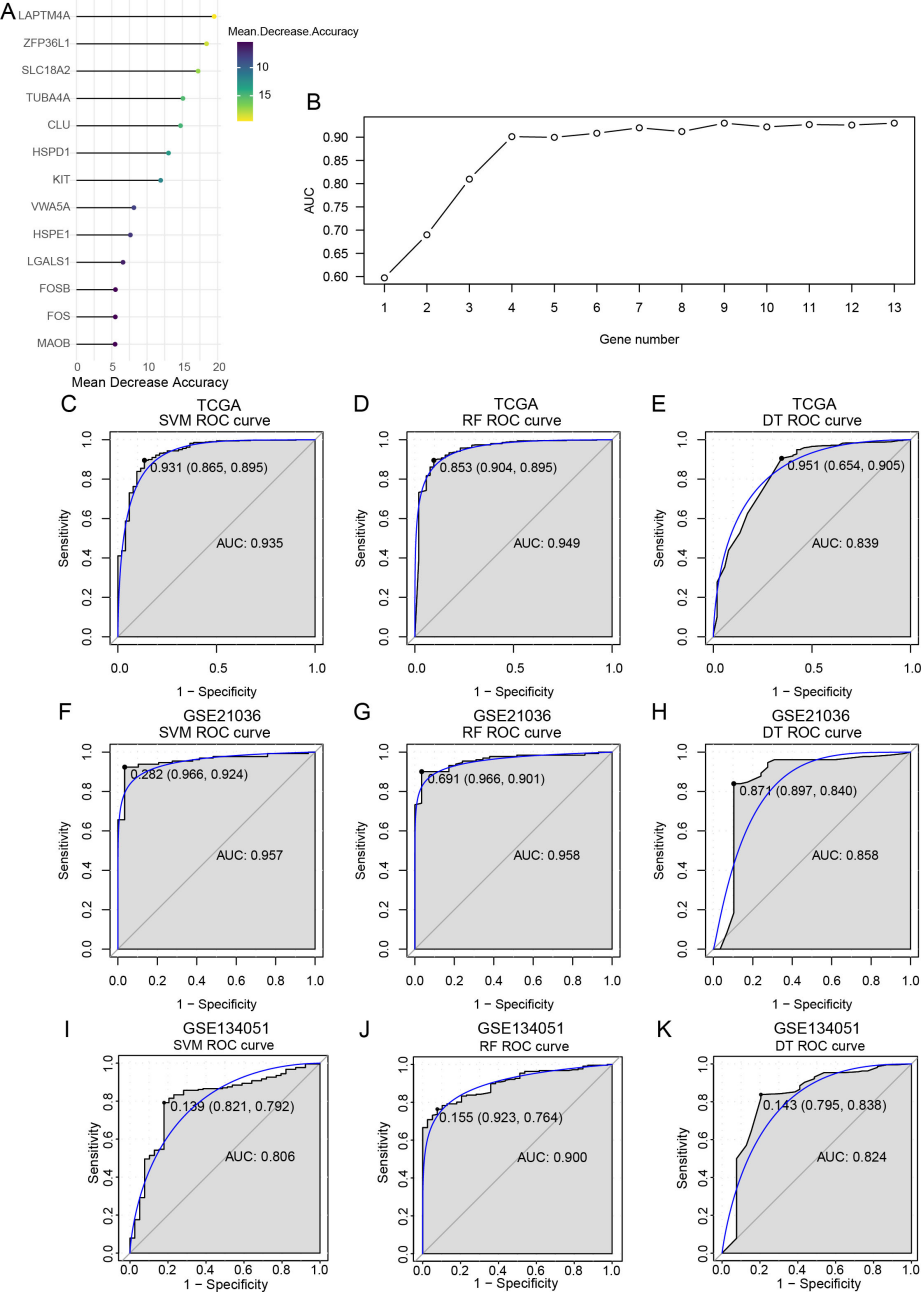
Construction of a prostate cancer diagnosis model. **(A)** Bar chart displaying the mean decrease in accuracy for a sequence of 13 differential genes, with a color gradient indicating the magnitude of decrease. **(B)** The performance of the random forest model measured by AUC at different numbers of top-ranked genes. The model was evaluated using 10-fold cross-validation. **(C)** ROC curve for the SVM model. **(D)** ROC curve for the RF model. **(E)** ROC curve for the DT model. **(F–H)** Prostate cancer diagnosis model validation in the GSE21034 dataset. **(I–K)** Prostate cancer diagnosis model validation in the GSE134051 dataset.

We employed machine learning algorithms to identify optimal NK cell-related diagnostic genes and subsequently developed diagnostic models. Through LASSO analysis, 13 core genes were ranked based on the average decrease in accuracy (**Figure 3A**). The results of the 10-fold cross-validation showed that the accuracy was highest when the number of genes was nine (**Figure 3B**). Based on this, the top nine core genes were considered optimal diagnostic genes. Significant expression correlations were observed among these genes (**Supplementary Figure 3A**). The top correlated pairs were HSPD1-HSPE1, VWA5A-KIT, VWA5A-ZFP36L1, VWA5A-CLU and LAPTM4A-CLU. PPI analysis indicated potential interactions among six of the genes, except for LAPTM4A, VWA5A, and ZFP36L1 (**Supplementary Figure 3B**). Subsequently, SVM, RF, and DT models were established according to the nine optimal diagnostic genes. The areas under the curve (AUC) of the SVM, RF, and DT models were 0.935, 0.949, and 0.839, respectively (**Figures 3C–E**). The sensitivities of the SVM, RF, and DT models were 86.5%, 90.4%, and 65.4%, respectively, while their specificities were 89.5%, 89.5% and 90.5%, respectively. We also verified the accuracy of these models using the independent datasets GSE21034 (**Figures 3F–H**) and GSE134051 (**Figures 3I–K**). In GSE21034, the AUC values of the SVM, RF, and DT models were 0.957, 0.985, and 0.858, respectively, whereas in GSE134051, the corresponding values were 0.806, 0.900, and 0.824. The sensitivity and specificity were > 75%. These results suggest that these NK cell related genes are associated with prostate cancer and may have potential utility in its early detection and diagnosis.

We also predicted potential lncRNA-miRNA-mRNA regulatory axes for the nine diagnostic genes and constructed a ceRNA network (**Supplementary Figure 4A**). This network included 9 DEmiRNAs, 7 upregulated and 2 downregulated in prostate cancer samples from the GSE21036 dataset, as well as 24 DElncRNAs, including 10 upregulated and 14 downregulated in prostate cancer from the TCGA dataset. Correlation analysis revealed the top five DElncRNAs significantly associated with the nine diagnostic genes: *LINC00665*, *LINC00839*, *GATA3-AS1*, *LINC01550*, and *LINC00997* (**Supplementary Figures 4B, C**). Real-time qPCR showed that *LINC00665* was significantly upregulated in prostate cancer tissue compared to adjacent normal tissues, consistent with findings from the TCGA dataset (**Supplementary Figure 4D**).

### 3.4 Drug sensitivity analysis

We identified nine drugs whose IC50 values showed significant differences between high and low expression groups of the nine diagnostic genes in TCGA prostate cancer (**Table 1**). These drugs include: AICAR (a MAPK activator), AZD.0530 (a Src kinase inhibitor), Bexarotene (a selective RXR agonist), BIRB.0796 (a p38 MAPK inhibitor), CCT007093 (a WIP1 inhibitor), DMOG (a HIF-1α activator), Imatinib (a multi-targeted tyrosine kinase inhibitor), KIN001.135, and Lapatinib (a dual tyrosine kinase inhibitor targeting EGFR and HER2). These results suggest that the expression levels of NK-related diagnostic genes may influence the sensitivity of prostate cancer cells to these compounds, providing a potential reference for individualized therapeutic strategies.

**Table 1:** P values for differences in IC50 of candidate drugs between high and low expression groups of 9 diagnostic genes in prostate cancer (TCGA).

Gene/drug	AICAR	AZD.0530	Bexarotene	BIRB.0796	CCT007093	DMOG	Imatinib	KIN001.135	Lapatinib
CLU	2.13863E-06	7.04866E-13	2.82367E-07	8.63613E-09	7.81885E-05	1.87387E-10	2.80572E-12	3.96672E-09	2.21424E-09
HSPD1	0.021283557	0.01899207	0.008905142	0.03172379	8.09549E-05	0.02173904	0.004040663	0.032366547	0.000544776
HSPE1	5.52652E-07	0.003306667	1.86186E-05	0.026174718	3.07493E-07	0.000530723	0.007094466	0.001736207	0.001916652
KIT	2.38E-11	5.03E-11	1.91E-08	1.66E-06	1.03E-08	1.63E-10	2.48E-09	9.30E-08	2.10E-07
LAPTM4A	3.28791E-05	0.00066703	0.000594212	0.015986968	0.001892858	0.008228876	8.97839E-07	6.12032E-06	0.000165102
SLC18A2	3.2159E-07	0.001172729	1.69422E-05	0.002085942	1.69048E-08	1.11073E-05	6.111E-07	0.000750067	4.67123E-05
TUBA4A	1.3275E-08	4.57305E-09	1.57683E-07	0.008316595	0.000361205	0.01182185	0.001202618	0.001761291	0.002306066
VWA5A	3.86523E-13	4.2198E-13	1.19127E-10	1.2593E-06	1.10772E-08	2.02102E-09	2.47133E-10	2.38076E-07	5.02283E-09
ZFP36L1	4.28737E-12	2.98523E-11	1.90985E-12	2.42248E-06	4.91646E-09	8.70408E-06	3.17691E-06	1.12692E-07	1.70176E-07

### 3.5 Expression verification of nine optimal diagnostic genes and KIT

**Figure 4:**
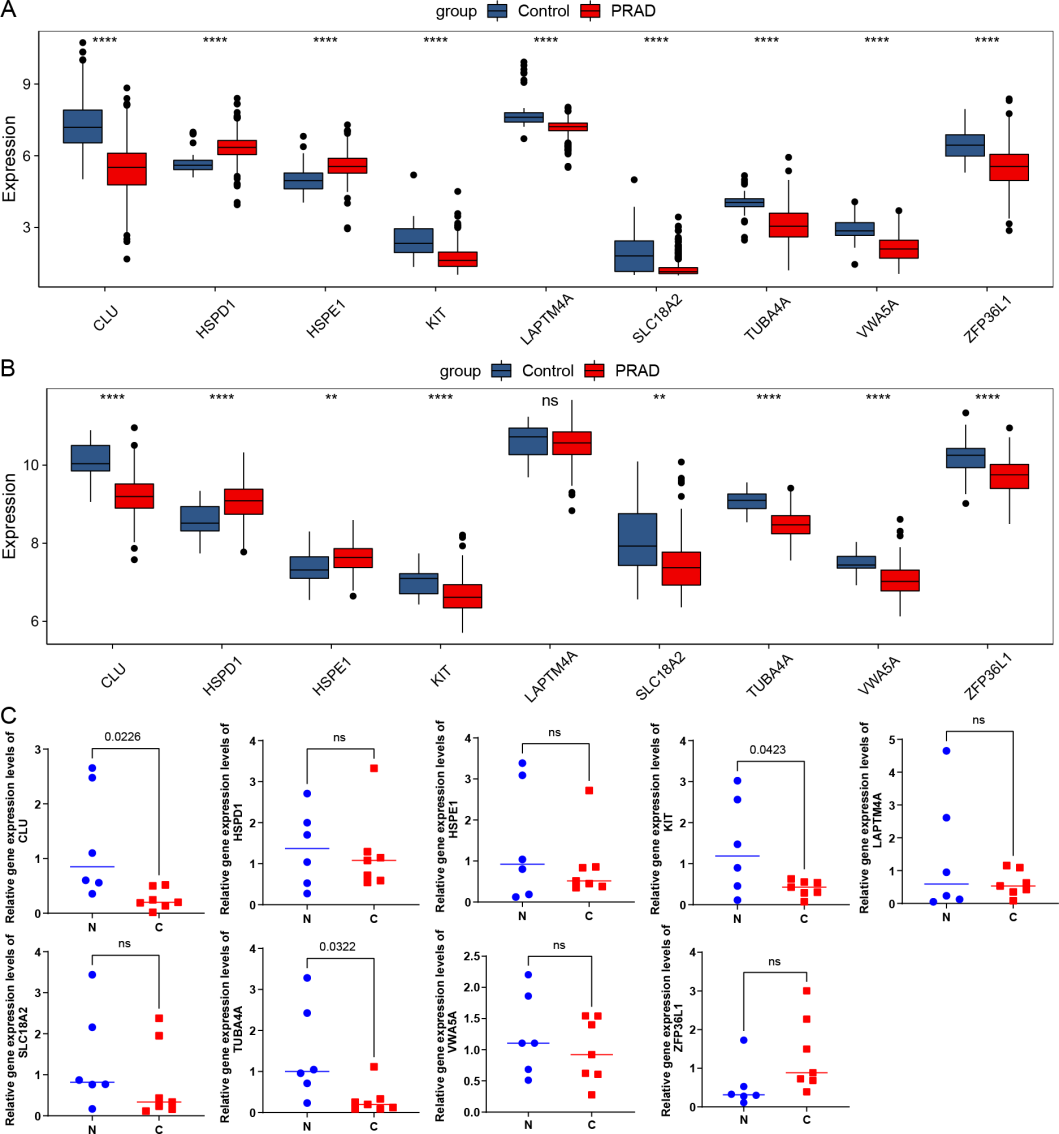
Verification of expressions for diagnostic genes. **(A)** Box plot showing the expression of diagnostic genes in prostate cancer and adjacent tissues in the TCGA dataset. **(B)** Box plot showing the expression of diagnostic genes in prostate cancer and adjacent tissues in the GSE21034 dataset. **(C)** Scatter plot showing the expression levels of diagnostic genes in prostate cancer and adjacent normal tissues measured by real-time qPCR. ns, not significant. **p-value < 0.01, ****p-value < 0.0001.

In the TCGA prostate cancer dataset, *HSPD1* and *HSPE1* were markedly upregulated in the prostate cancer group, whereas *CLU*, *KIT*, *LAPTM4A*, *SLC18A2*, *TUBA4A*, *VWA5A*, and *ZFP36L1* were markedly downregulated in the prostate cancer group compared to the control group (**Figure 4A**). Except for *LAPTM4A*, the expression of the other genes (or their protein levels) in the GSE21034 dataset and Human Protein Atlas database was consistent with findings from the TCGA prostate cancer dataset (**Figure 4B**; **Supplementary Figure 5**). Subsequently, tumor and adjacent normal tissue samples from prostate cancer patients were collected to validate the expressions of nine diagnostic genes using real-time qPCR. The results showed that *CLU*, *KIT* and *TUBA4A* were significantly differentially expressed between the two groups (**Figure 4C**). The expression levels of the nine optimal diagnostic genes in each cell cluster are shown in **Supplementary Figure 6**.

### 3.6 Validation of the co-expression of KIT and CD56 in prostate cancer tissues

**Figure 5:**
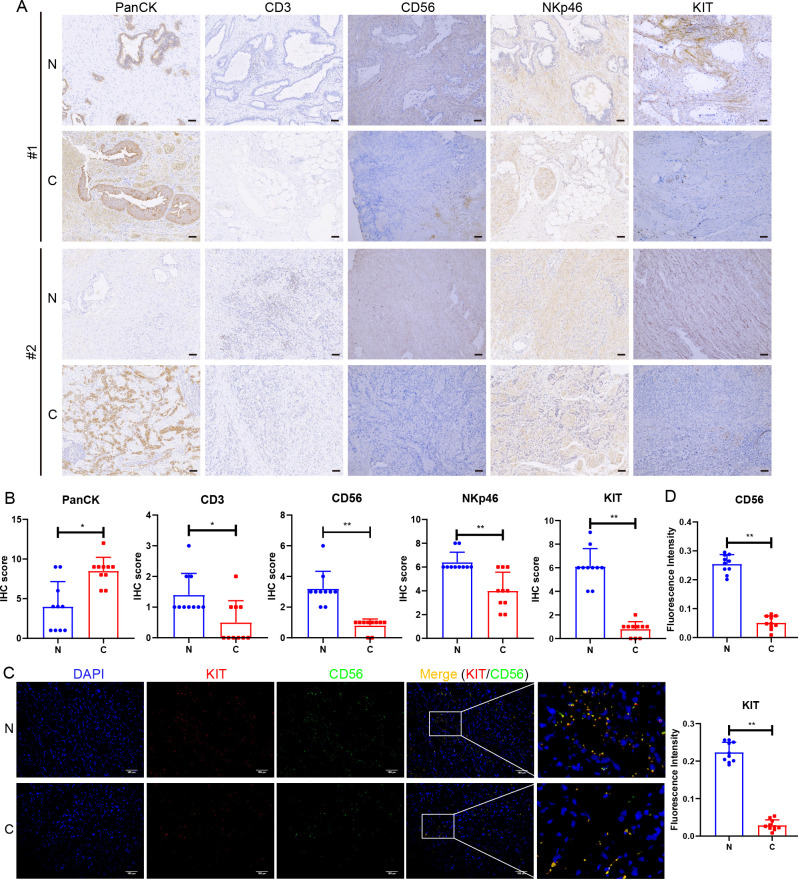
The protein expressions of KIT and CD56 in prostate cancer and adjacent tissues. **(A, B)** The expression of PanCK, CD3, NKp46, KIT and CD56 in prostate cancer and adjacent tissues detected by IHC assay. bar = 10 μm. **(C, D)** The expression of KIT (red) and CD56 (green) in prostate cancer and adjacent tissues detected by IF assay. C, prostate cancer tissues; N, adjacent tissues. bar = 100 μm. **p-value < 0.01, *p-value < 0.05.

Previous research has reported that upregulated KIT expression enhances the adhesion of CD56(bright) NK cells to membrane-bound stem cell factor (25). This evidence suggests a potential functional link between KIT and NK cell activity. Therefore, KIT was selected for subsequent analyses. We first performed immunohistochemical staining for PanCK as a pan-tumor epithelial marker to delineate tumor regions. As expected, PanCK protein expression was significantly increased in prostate cancer tissues compared with adjacent tissues (**Figures 5A, B**). Subsequently, immunohistochemical analyses were conducted using CD56, a commonly used NK cell marker, and NKp46, a more NK cell-specific marker, together with CD3 to assess T-cell distribution. The results showed that CD56- and NKp46-positive cells were both reduced in prostate cancer tissues, while CD3-positive cells exhibited limited staining areas. These findings indicated a reduced infiltration of NK cells in cancer tissues (**Figures 5A, B**). IF analysis revealed the co-expression of KIT and CD56 in both prostate cancer and adjacent normal tissues and further confirmed their decreased expression levels in cancer tissues (**Figures 5C, D**).

### 3.7 Overexpression of KIT can enhance the ability of NK cells to kill cancer cells

**Figure 6:**
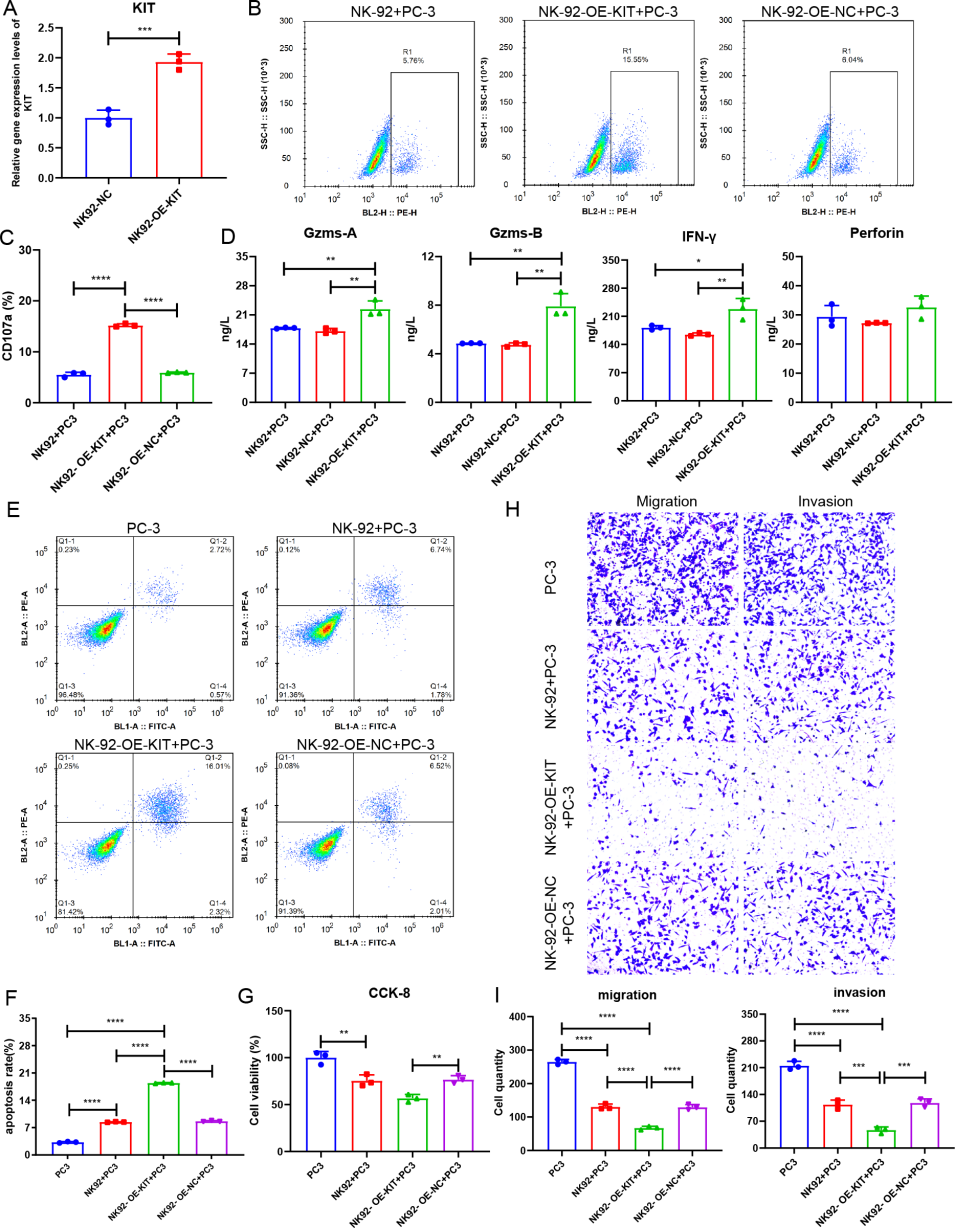
Investigating the impact of KIT overexpression on PC-3 cell migration, invasion, and NK cell cytotoxicity. **(A)** Real-time qPCR validation of KIT overexpression in NK-92 cells. **(B, C)** Flow cytometry analysis of CD107a expression, a marker of NK cell degranulation, after co-culturing KIT-overexpressing NK-92 cells with PC-3 cells for 48 h. **(D)** Effect of KIT overexpression on the secretion of key NK cell cytotoxicity-related factors, including IFN-γ, Granzyme A (Gzms-A), Granzyme B (Gzms-B), and Perforin, after co-culturing KIT-overexpressing NK-92 cells with PC-3 cells for 48 h. **(E, F)** Apoptosis of PC-3 cells was assessed by flow cytometry after co-culture with KIT-overexpressing, vector control, or untreated NK-92 cells for 48 h. **(G)** Cell viability of PC-3 cells was evaluated by CCK-8 assay with KIT-overexpressing, vector control, or untreated NK-92 cells for 48 h. **(H, I)** The effects of KIT-overexpressing NK-92 cells on the migration and invasion of PC-3 cells were evaluated using Transwell assays. Quantification was based on the number of PC-3 cells that migrated or invaded through the membrane pores. *p-value < 0.05, **p-value < 0.01, ***p-value < 0.001, ****p-value < 0.0001.

To investigate the role of KIT in regulating NK cell function, we generated *KIT*-overexpressing NK-92 cells (**Figure 6A**). Next, we co-cultured *KIT*-overexpressing NK-92 cells with PC-3 prostate cancer cells and assessed degranulation by measuring CD107a expression via multiparameter flow cytometry (**Figures 6B, C**). KIT overexpression led to a higher percentage of CD107a^+^ NK-92 cells following co-culture, suggesting enhanced activation and degranulation in response to cancer cell stimulation (15.21 ± 0.30 vs 5.95 ± 0.09). Consistently, ELISA results of the co-culture supernatants showed that overexpression of KIT significantly enhanced the secretion of granzyme A (Gzm-A), granzyme B (Gzm-B), and interferon-γ (IFN-γ), reflecting increased secretion of cytotoxic effectors as a functional consequence of enhanced degranulation (**Figure 6D**). Furthermore, compared to PC-3 cells cultured alone, co-culture with NK-92 cells significantly increased apoptosis (**Figures 6E, F**) and decreased cell viability (**Figure 6G**) of PC-3 cells. Moreover, Transwell assays demonstrated that co-culture with NK-92 cells significantly suppressed the migration and invasion (**Figures 6H, I**) of PC-3 cells. These effects were further amplified when KIT was overexpressed in NK-92 cells (**Figures 6E–I**). Collectively, these findings demonstrate that KIT overexpression enhances the cytotoxic activity of NK cell effector functions and promotes NK cell-mediated anti-tumor effects against prostate cancer cells.

Using the CB-Dock2 online tool, we predicted the optimal binding scores between KIT and nine candidate compounds. The results showed that DMOG and the other eight candidate drugs all exhibited predicted binding scores lower than -5.0 with KIT, indicating favorable binding potential (**Supplementary Table 2**). Among these compounds, lapatinib demonstrated the lowest predicted binding energy (Vina score = -11.5), suggesting a relatively strong potential interaction with KIT.

## 4 Discussion

Immune cells within the TIME play crucial roles in both immune surveillance and immune evasion. Previous studies have demonstrated that elevated levels of CD56^+^ NK cells in peripheral blood are associated with a favorable prognosis in patients with prostate cancer (26), and NK cell activity may also possess diagnostic potential (27). Inspired by these studies, we attempted to identify NK cell related genes and develop candidate diagnostic biomarkers for prostate cancer through the integrated analysis of single-cell and bulk RNA-sequencing in our study.

In our study, we identified a nine-gene NK cell-related diagnostic signature (*HSPD1*, *HSPE1*, *CLU*, *KIT*, *LAPTM4A*, *SLC18A2*, *TUBA4A*, *VWA5A*, and *ZFP36L1*) that exhibited robust diagnostic performance in different public cohorts (AUC > 0.8), partially mitigating concerns regarding potential overfitting associated with the multi-step feature selection strategy and offering novel insights into the link between NK cell dysfunction and prostate cancer progression. Notably, in the GSE134051 validation cohort, the diagnostic performance of the current model was within a comparable range to the previously reported PSA-based screening performance (AUC = 0.837) from the same cohort (28). Tissue-based transcriptomic signatures may provide diagnostic information complementary to conventional serum biomarkers such as PSA. Several genes in this signature are known to influence NK cell function. For example, CLU (clusterin), a stress-responsive glycoprotein, has been reported to synergize with IL-2 to promote NK cell proliferation and IFN-γ production (29). KIT, a member of the type III receptor tyrosine kinase family, can regulate NK cell activity and responsiveness to stem cell factor (SCF) (30). Despite the unclear roles of ZFP36L1 (zinc finger protein 36 like 1), LAPTM4A (lysosomal protein transmembrane 4 alpha), and TUBA4A (tubulin alpha 4a) in NK cells, their potential association with macrophage differentiation or polarization indicates a broader connection with innate immune responses (31–33). Studies have shown that NK cells exhibit tissue-specific phenotypes and that the tumor microenvironment can reshape NK cell transcriptional programs (34, 35). The identified genes may capture transcriptional features associated with NK-related immune states and broader tumor microenvironment interactions, rather than being restricted to classical NK cell marker-defined biology.

Beyond their potential roles in NK cells, these genes are also associated with cancer development, particularly in prostate cancer. HSPD1 (Heat shock protein family D member 1) and HSPE1 (heat shock protein family E member 1), both mitochondrial chaperones, have been implicated in tumor cell proliferation, apoptosis, and metastasis (36–38). Kumar et al. found that HSPD1 regulates IL-8-mediated apoptosis of prostate cancer cells both *in vivo* and *in vitro* (39). Abnormal expression of *HSPE1* has been reported to be associated with the development of multiple tumors, including lung cancer, breast cancer, and acute myeloid leukemia (40–42). A recent study has shown that HSPE1 can be used as an indicator of prostate cancer invasiveness and is a potential risk factor for prostate cancer (43). In addition, CLU mediates the progression of multiple cancers, including pancreatic, liver, bladder, breast, and prostate cancers (44–47). Bertacchini et al. found that CLU regulates the expression of PHLPP1 in prostate cancer via miR-190-dependent regulation, thus stabilizing AKT2 phosphorylation and transforming a low-migration phenotype into a high-migration phenotype (48). The SLC18A2 (solute carrier family 18 member 2) was also associated with prostate cancer aggressiveness, it had been found to be hypermethylated in prostate cancer and can be used as a biomarker for the diagnosis and prognosis of prostate cancer (49, 50). ZFP36L1 and VWA5A have also been linked to tumor suppression in various cancers (51, 52). Knockdown of *LAPTM4A* suppresses glioma invasion and EMT marker expression (53). In a study on papillary thyroid carcinoma, KIT was upregulated by CTC/miR-146 axis, which in turn inhibits cancer cell proliferation, migration, and invasion (54). Together, these findings support the diagnostic and functional relevance of this NK cell-related gene signature in cancer. Furthermore, our analyses revealed significant co-expression patterns and potential functional interactions among these diagnostic genes, suggesting that they may converge on shared pathways influencing NK cell functions in prostate cancer.

Particularly, our study has focused on the role of the *KIT* gene in prostate cancer. Previous research has shown that KIT is a key regulator of the activation status and function of CD56^bright^ NK cells, and its low expression may be associated with NK cell dysfunction or functional exhaustion (55). In prostate cancer, NK cell cytotoxicity is markedly impaired, characterized by an exhausted NK phenotype (56), which may contribute to immune evasion and tumor progression (57). In our study, we found that *KIT* overexpression significantly enhanced the cytotoxic function of NK cells, as evidenced by increased secretion levels of cytokines (such as IFN-γ, Gzms-A, Gzms-B, and Perforin) and improved degranulation ability of NK cells. Further experiments demonstrated that overexpression of *KIT* significantly enhanced the cytotoxicity of NK-92 cells against prostate cancer cells and inhibited cancer cell migration and invasion. These findings suggest that KIT may enhance anti-tumor immunity in prostate cancer by boosting NK cell cytotoxicity.

Through drug sensitivity analysis, we identified nine compounds whose IC50 values were significantly associated with the expression levels of the diagnostic genes in prostate cancer. Among them, AICAR induces apoptosis in prostate cancer cells by activating AMPK (58) and has been shown to enhance radiosensitivity (59). Bexarotene may overcome multidrug resistance in advanced prostate cancer (60) and sensitize CRPC cells to docetaxel (61). In addition, molecular docking analysis suggested relatively high predicted binding affinities between KIT and several candidate compounds. Although these observations are based on in silico analyses, they may provide preliminary hypotheses for future studies investigating KIT-related candidate compounds in prostate cancer models. Emerging evidence suggests that drug sensitivity may be influenced not only by tumor-intrinsic mechanisms but also by immune cell–related regulatory pathways (62). Therefore, further experimental studies will be required to determine whether any of the prioritized compounds modulate KIT activity and whether such modulation has measurable effects on tumor phenotypes and/or NK cell-associated antitumor responses.

Despite these promising findings, our study has some limitations. First, the limited sample size in the scRNA-seq dataset may restrict the generalizability and reproducibility of the NK cell related genes identified. Second, our model was derived and validated using bulk RNA-seq data. While bulk data are widely used and clinically accessible, they cannot definitively assign the signature’s expression signal to specific cell types. Our scRNA-seq and deconvolution correlation analyses suggested that the signature captures NK cell biology, but their association with NK cell biology requires further experimental validation. Third, the genes examined in our study were limited to NK cell related genes, and the TIME was highly spatially heterogeneous. Therefore, the diagnostic capability of NK cell related genes is limited. Future studies could adopt more advanced analytical methods, such as using neural network models in conjunction with spatial omics and imaging data, to validate NK cell related genes (63). This approach may improve the diagnosis of prostate cancer and enhance our understanding of the spatial heterogeneity of the tumor microenvironment. Fourth, platform differences between scRNA-seq and bulk RNA-seq may introduce subtle batch-related effects that could influence gene detectability. Finally, the *in vitro* validation was performed exclusively using NK-92 and PC-3 cells. As NK-92 cells are transformed and IL-2 dependent, and PC-3 cells represent a poorly differentiated and highly metastatic prostate adenocarcinoma phenotype (64), these single cell lines cannot fully recapitulate primary NK cell biology and capture the biological heterogeneity of different prostate cancer subtypes.

## 5 Conclusion

In summary, our study developed a diagnostic model for prostate cancer based on nine NK cell related genes, including *HSPD1*, *HSPE1*, *CLU*, *KIT*, *LAPTM4A*, *SLC18A2*, *TUBA4A*, *VWA5A*, and *ZFP36L1*. These genes collectively offer potential for early detection and diagnosis. Notably, KIT is significantly downregulated in prostate cancer tissues, and its overexpression enhances NK-92 cell cytotoxicity while inhibiting PC-3 cell migration and invasion. Therefore, KIT may be a promising candidate worthy of further studies evaluating its potential therapeutic relevance. Future studies should validate the roles and diagnostic potential of these genes across additional cell lines or primary cells, *in vivo* models (gain-of-function and loss-of function), and larger clinical cohorts. Moreover, exploring the correlation between the identified drugs and diagnostic genes represents an important avenue for advancing prostate cancer therapy.

## Data Availability

The data presented in this study were obtained from publicly available databases: GEO database, accession number GSE153892, GSE21034, GSE134051; UCSC Xena, TCGA-PRAD.
