# Supplementary material for: A diagnostic signature derived from NK cell related genes in prostate cancer: insights from integrated scRNA-seq and bulk RNA-seq analyses with functional validation of KIT
Source: Front Immunol. 2026 Jun 3;17:1692792. doi: 10.3389/fimmu.2026.1692792 (PMC13272176; doi:10.3389/fimmu.2026.1692792)
Supplement: Supplementary file 1 [file Table1.docx]

***Supplementary Material***

**Supplementary Figures and Tables**

**Supplementary Figures**


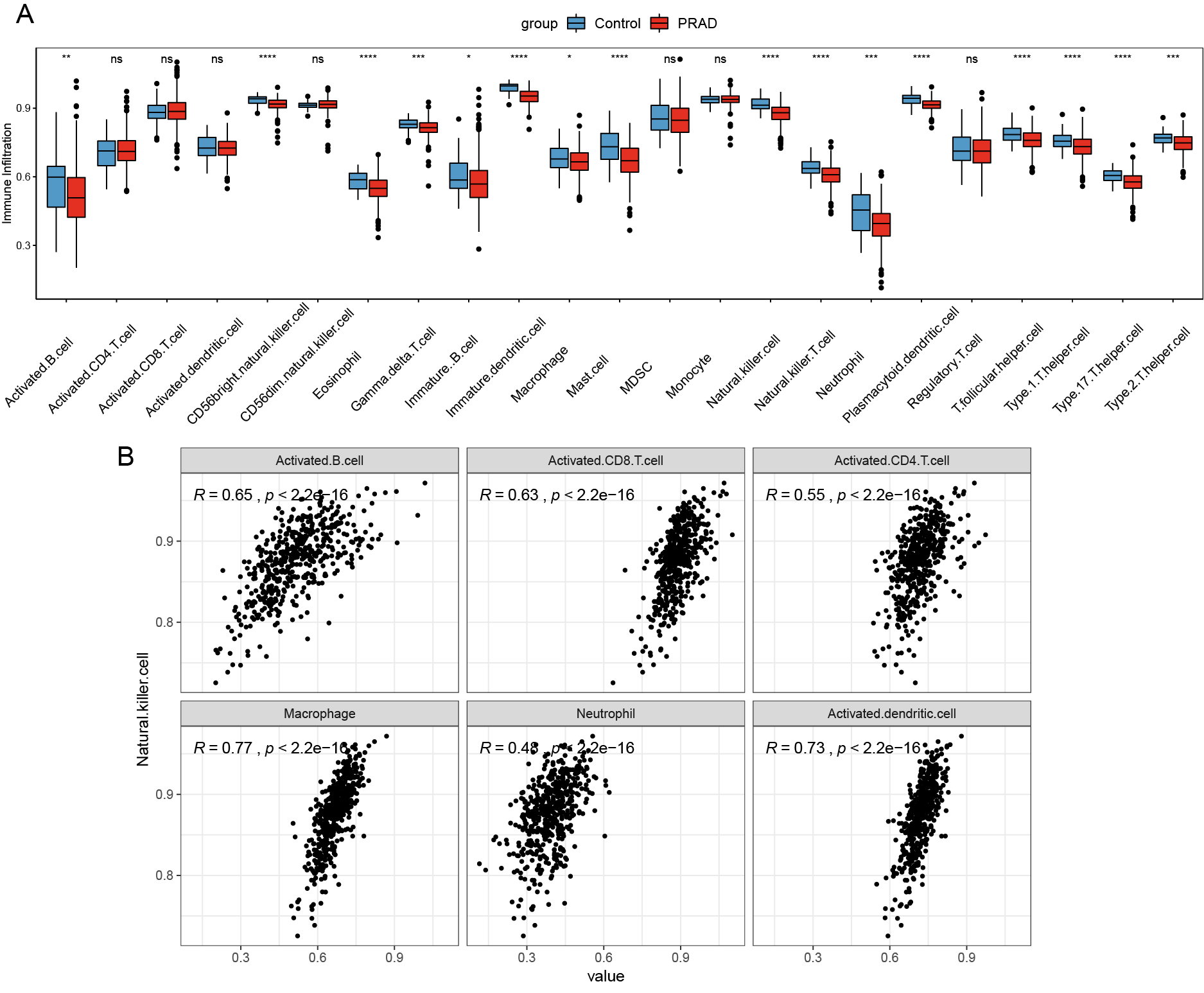


**Supplementary Figure 1 Assessment of cell infiltration in the tumor microenvironment of prostate cancer**

(A) Boxplots of differences in 28 cell types between prostate cancer and normal control, highlighting significant differences with statistical annotation. * p-value < 0.05, ** p-value < 0.01, *** p-value < 0.001, **** p-value < 0.0001. (B) Scatter plots showing the correlation between NK cells and other immune cell types, including activated B cells, activated CD8 T cells, activated CD4 T cells, macrophages, neutrophils, and activated dendritic cells.


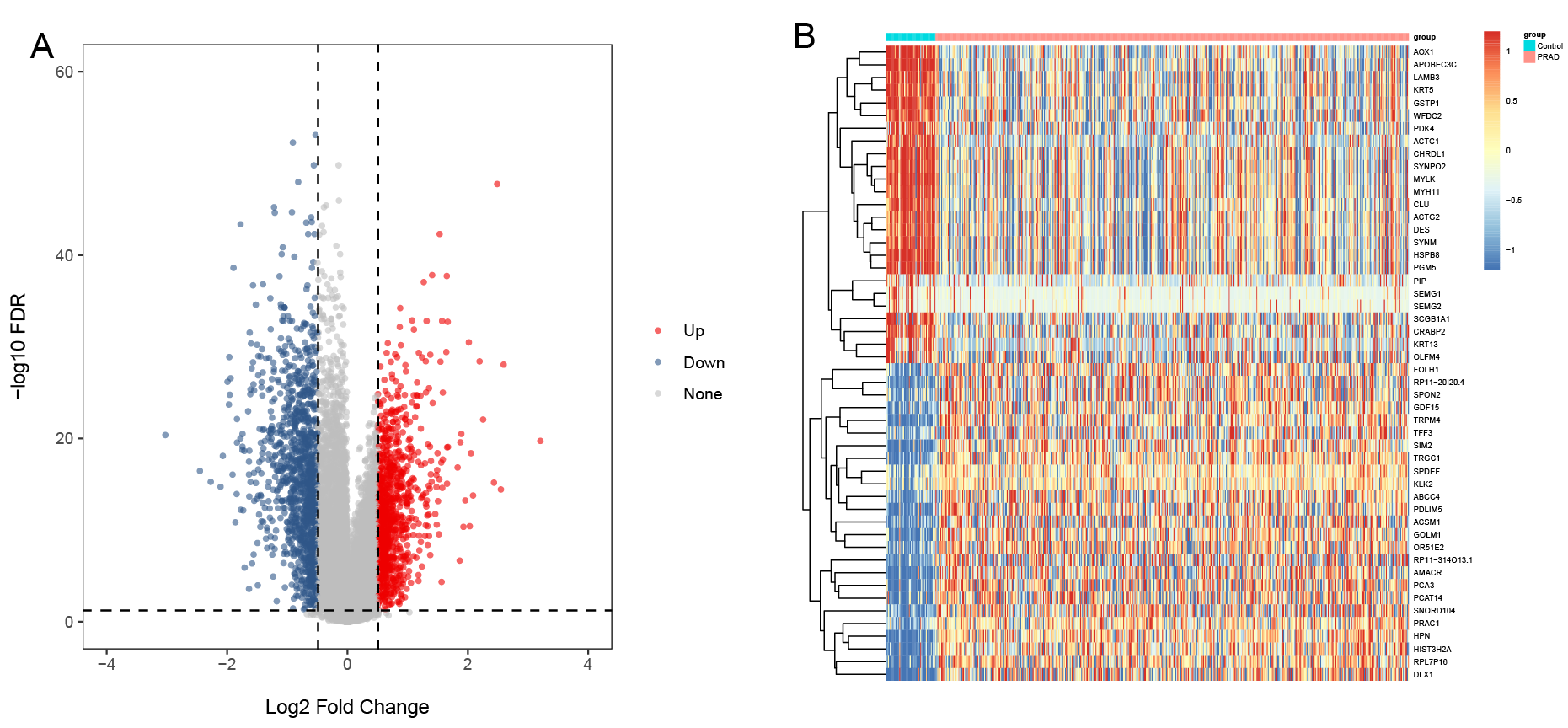


**Supplementary Figure 2 DEGs between prostate cancer and normal control in TCGA**

(A) Volcano plot of DEGs between prostate cancer and normal control in TCGA. (B) [Heat](file:///D:\360Downloads\Youdao\Dict\7.2.0.0703\resultui\dict\?keyword=heat)[map](file:///D:\360Downloads\Youdao\Dict\7.2.0.0703\resultui\dict\?keyword=map) of Top 25 DEGs between prostate cancer and normal control in TCGA.


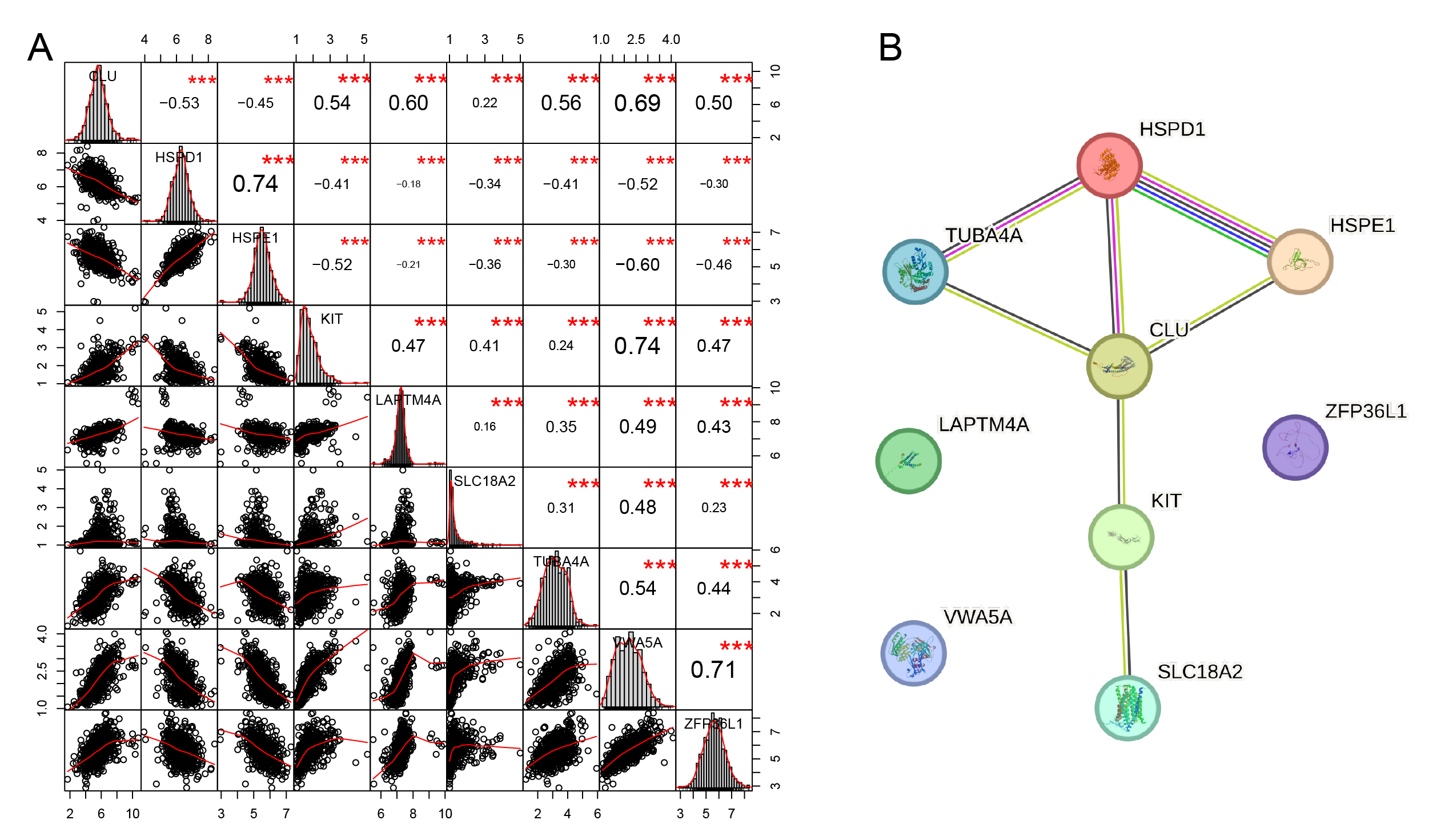


**Supplementary Figure 3 Diagnostic genes association analysis**

(A) The correlations between nine diagnostic genes. (B) Protein-protein internation analysis between diagnostic genes. *** p-value < 0.001.


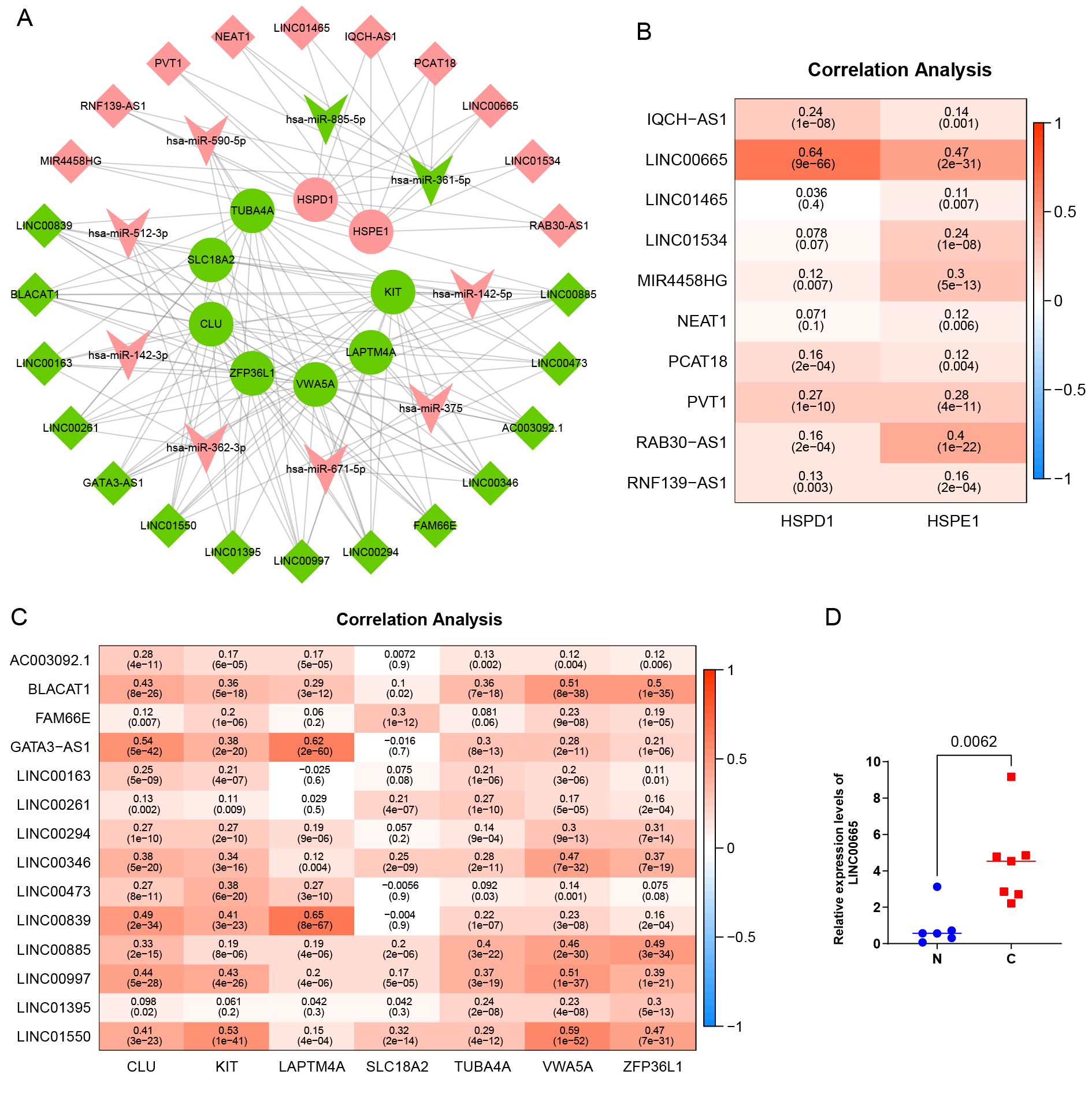


**Supplementary Figure 4 Establishment of ceRNA regulatory network in prostate cancer**

(A) ceRNA regulatory network diagram. The circle, squares and inverted triangle represent genes, lncRNAs and miRNAs, respectively. Red color represents up-regulated and green color represents down-regulated. (B) Heatmap of the correlation analysis between up-regulated lncRNAs and up-regulated mRNAs. (C) Heatmap of the correlation analysis between down-regulated lncRNAs and down-regulated mRNAs. (D) Real-time qPCR analysis of *LINC00665* expression in adjacent normal (N, n = 6) and prostate cancer (C, n = 7) tissues.


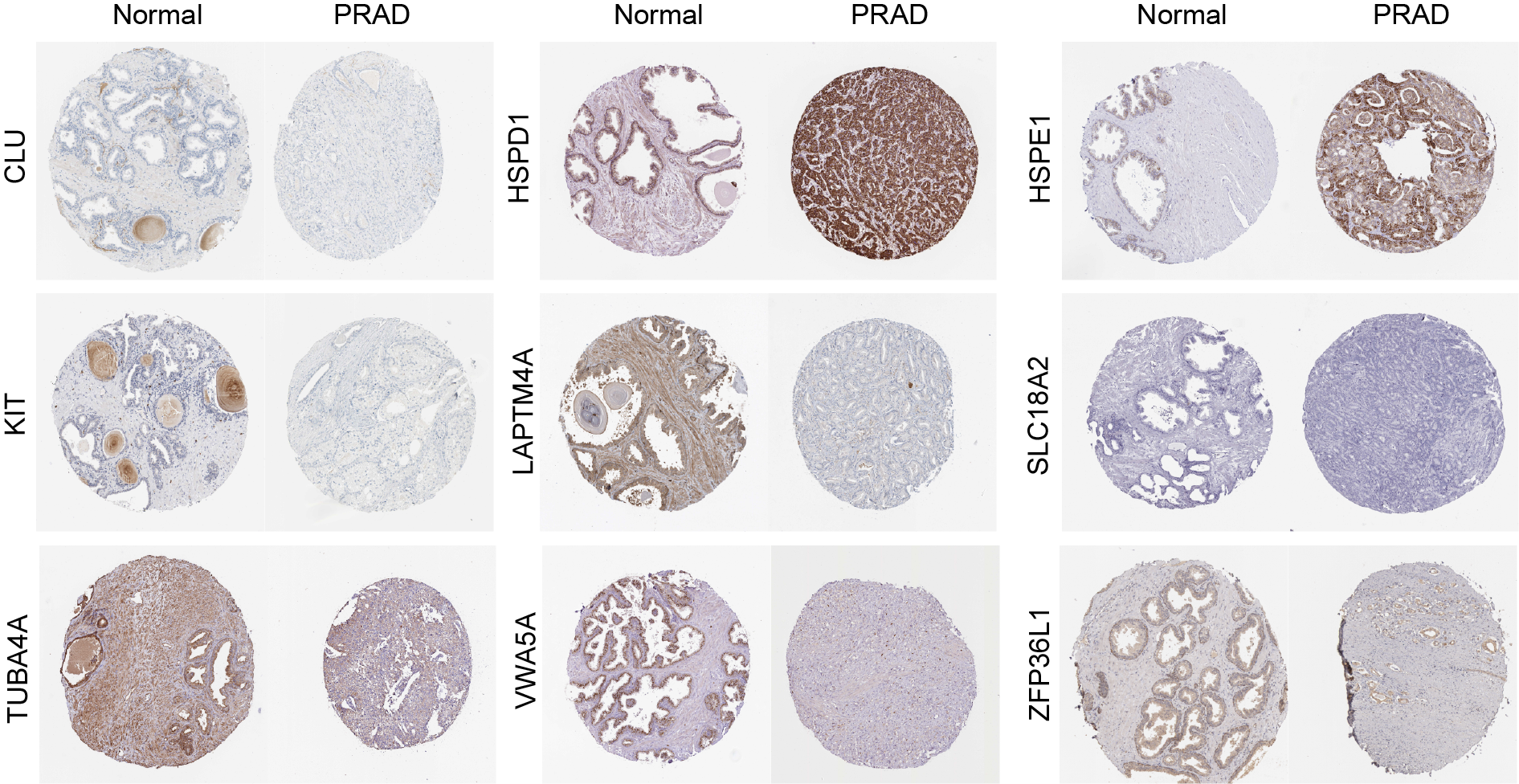


**Supplementary Figure 5 Expression level of the nine proteins in the Human Protein Atlas database**

Normal: normal tissues; PRAD: prostate cancer tissues.


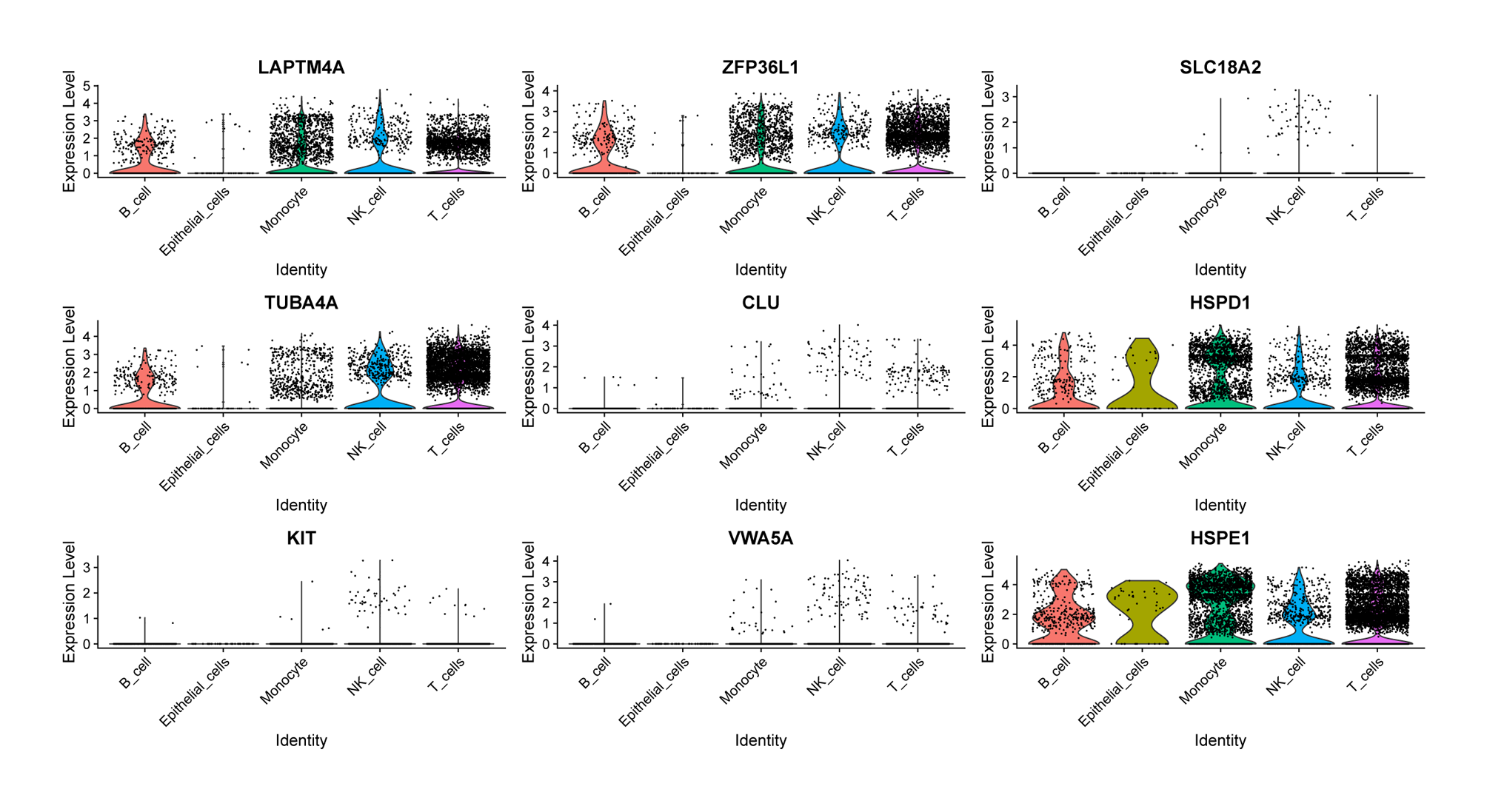


**Supplementary Figure 6 The expression level of the nine genes in each cell cluster**

**Supplementary Table**

**Supplementary Table 1 Real time qPCR primer sequence**

| **primer** | **sequence (5'to3')** |
| --- | --- |
| GAPDH-F | GGAGCGAGATCCCTCCAAAAT |
| GAPDH-R | GGCTGTTGTCATACTTCTCATGG |
| ACTB-F | CATGTACGTTGCTATCCAGGC |
| ACTB-R | CTCCTTAATGTCACGCACGAT |
| CLU-F | CCATGTTCCAGCCCTTCCTT |
| CLU-R | GACAAGATCTCCCGGCACTT |
| HSPD1-F | AGTCAAGGCTCCAGGGTTTG |
| HSPD1-R | GCATGGCATCGTCTTTGGTC |
| KIT-F | TGTGCCTGTTGTGTCTGTGT |
| KIT-R | GTCACCGTGATGCCAGCTAT |
| LAPTM4A-F | TCCATGCCAGCTGTCAACAT |
| LAPTM4A-R | GGAATCAGCCAACCCACTTG |
| SLC18A2-F | CTGCCTCCATCTCAGACAGC |
| SLC18A2-R | GGGACAGTCGGAAGGAACAG |
| TUBA4A-F | TGGACCACAAGTTCGACCTG |
| TUBA4A-R | CATAGGAGTCGATGCCCACC |
| VWA5A-F | CTCTGCAGCCTGTGGTAGAG |
| VWA5A-R | TCCTGTTGTCTCTGCTGCTG |
| ZFP36L1-F | TCCACCAGAACCAGCTCCT |
| ZFP36L1-R | TGTAGCGGCTGGAGTTGAC |
| LINC00665-F | AGGTACGACATTTGGAGGCC |
| LINC00665-R | CATGGTAGTCGATCCGCTGT |

**Supplementary Table 2 CB-Dock2 results of KIT and predicted**

| **Drug** | **CID** | **Vina score** | **Cavity volume (Å3)** | **Center (x, y, z)** | **Docking size (x, y, z)** |
| --- | --- | --- | --- | --- | --- |
| AICAR | 17513 | -6.1 | 3051 | 42, 11, 45 | 25, 25, 25 |
| AZD.0530 | 10302451 | -9 | 3051 | 42, 11, 45 | 25, 25, 25 |
| Bexarotene | 82146 | -7.8 | 215 | 34, 5, 62 | 22, 22, 22 |
| BIRB.0796 | 156422 | -9.3 | 3051 | 42, 11, 45 | 27, 27, 27 |
| CCT007093 | [2314623](https://pubchem.ncbi.nlm.nih.gov/compound/2314623) | -8.3 | 3051 | 42, 11, 45 | 22, 22, 22 |
| DMOG | [560326](https://pubchem.ncbi.nlm.nih.gov/compound/560326) | -4.7 | 3051 | 42, 11, 45 | 25, 25, 25 |
| Imatinib | [5291](https://pubchem.ncbi.nlm.nih.gov/compound/5291) | -10.6 | 3051 | 42, 11, 45 | 26, 26, 26 |
| KIN001.135 | [11626927](https://pubchem.ncbi.nlm.nih.gov/compound/11626927) | -8.5 | 3051 | 42, 11, 45 | 24, 24, 24 |
| Lapatinib | [208908](https://pubchem.ncbi.nlm.nih.gov/compound/208908) | -11.5 | 3051 | 42, 11, 45 | 26, 26, 26 |
